# Associations of socio-demographic factors with adiposity among immigrants in Norway: a secondary data analysis

**DOI:** 10.1186/s12889-020-08918-9

**Published:** 2020-05-24

**Authors:** Samera Azeem Qureshi, Melanie Straiton, Abdi A. Gele

**Affiliations:** 1 Unit for Migration & Health, Norwegian Institute of Public Health (NIPH), P.O.Box 222, 0213 Oslo, Skøyen Norway; 2 Department of Mental Health and Suicide, Norwegian Institute of Public Health (NIPH), P.O.Box 222, 0213 Oslo, Skøyen Norway

**Keywords:** Obesity, Immigrants, Public health, Norway, Odds ratio

## Abstract

**Background:**

Obesity is becoming an important public health challenge, especially among immigrants coming from low and middle income to high-income countries. In this study we examined the relationship between overweight/obesity and various socio-demographic indicators among different immigrant groups in Norway.

**Methods:**

We used data from the *Living Conditions Survey among Immigrants 2016*, conducted by Statistics Norway. Our study sample included 4194 immigrants from 12 different countries. Participants were asked about a number of topics including health, weight, height, demographic factors, length of residence and employment. We ran logistic regression analysis to determine the odds ratio (OR) of the associations between socio-demographic factors with adiposity among immigrants.

**Results:**

Approximately 53% of the sample was overweight/obese. There was a significant difference in overweight/obesity by gender, age, country of origin and marital status. Overall immigrant men were almost 52% more likely to be overweight/obese than women. Women from Somalia had the highest odds (13.1; CI: 7.4–23.1) of being overweight/obese, followed by Iraq (8.6; CI: 4.9–14.9), Pakistan (7.5; CI: 4.2–13.4), Kosovo (7.0; CI: 4.1–12.1), and Turkey (6.8; CI: 4.0–11.6) as compared to the women from Vietnam (reference). Whereas men from Turkey had the highest odds (5.2; CI: (3.2–8.3)) of being overweight/obese, followed by Poland (4.2; CI: 2.7–6.1), Bosnia (4.1; CI: (2.6–6.5) and Kosovo (3.9; CI: 2.5–6.1). The odds for obesity increased with age and odds were highest in the eldest group 45–66 years (4.3; CI: 3.2–5.8) as compared to reference group16–24 years. The odds of being overweight/obese was higher among married (1.6; CI: 1.3–1.9) and divorced/separated/widowed (1.5; CI: 1.1–2.0) as compared to singles. Education, employment status, physical activity and length of residence were not associated with the odds of being overweight/obese.

**Conclusion:**

The findings of this study call attention to the importance of a greater understanding of the processes leading to obesity among certain immigrant groups in Norway. Moreover, there is a need for culturally adapted prevention strategies targeting immigrant men and women with high rates of overweight/obesity.

## Background

Obesity is a reversible predisposing factor and a serious threat for the development of chronic diseases such as cardiovascular disease (CVD), type 2 diabetes, cancer, hypertension and coronary heart disease [1–3]. Obesity is also linked to higher mortality and higher risk of mental health problems, musculoskeletal problems, and a general lower quality of life [2]. Despite being a leading cause of death, approximately 2 billion individuals are affected by obesity worldwide [4], and the number of overweight/obese individuals continue to grow with many finding it difficult to maintain a ‘normal’ weight in today’s largely obesogenic environment.

Prevalence of obesity is on the rise not only in high-income countries (HICs) but also in low- and middle-income countries (LMIC) [5–10]. As compared to 10 or 20 years ago, a much larger part of European populations are immigrants [4]. From 2000 to 2015, globally international migration increased by 41%, with over 244 million people now living in a country other than where they were born [11]. The population of immigrants in Europe, born outside the EU-states increased from 2,48 million in 2010 to 3,22 million in 2018 [12].

Findings from previous studies suggest that immigrants from LMIC have much healthier body weights and diet upon arrival to HICs, than the local community [13, 14]. However, some immigrant groups are at a higher risk of becoming overweight/obese [15–19]. Migration from LMIC to HIC profoundly affects lifestyle, in particular dietary habits, nutrient intake, and physical activity as a result of both westernization and urbanization [20–24]. Immigrants adapt to the more refined high energy density diet of the HIC, rich in fat and sugars. This combined with physical inactivity, interaction between genetic susceptibility and environmental factors (i.e. diet, smoking and exercise), psychological stress, immune-inflammatory changes, inequalities in access to and lower quality of health care, inappropriate management and the under detection of morbidities [23–25] all contribute to increase in weight.

Another important contributing factor being that healthy foods such as vegetables and fresh fruits or organic food is much more expensive than high energy density food, such as sweets or fats [26–28]. It is established that socio-economic factors play a considerable role in food choices [29], leading to an unhealthy weight gain and a greater obesity risk 10–15 years after migration [13]. In addition to lifestyle factors, socio-demographic factors such as sex, age, education, income level, marital status and physical activity affect body composition [30–32]. Increasing age, low income, low education and sedentary lifestyle are related to high BMI [30, 33]. Furthermore, published literature identifies global migration phenomenon as a social determinant of health [34].

According to a report by the Norwegian Institute of Public Health (NIPH), the prevalence of obesity among middle-aged (40–45 years) men was 25% and 20% in women in Norway [35]. Immigrants make up almost 18% (979,254) of Norway’s total population [36] however, few studies have examined obesity among immigrants in Norway [21, 37, 38], and to our knowledge no prior study has looked at the relationship between overweight/obesity and socio-demographic factors among different immigrant groups. In this study, we aim to fill this research gap by assessing the relationship between overweight/obesity and various sociodemographic indicators such as age, gender, education, marital status etc. among different immigrant groups in Norway.

## Methods

### Data source

This study is based on data from a cross-sectional population-based survey conducted by Statistics Norway; “Living Conditions Survey among Immigrants 2016 (LKI_2016)” in 2015/16 [36, 39]. The survey covered a number of topics including demographic factors, family, housing, employment and the working environment, social contact, discrimination, Norwegian language proficiency, attitudes and values and health. Many of the questions are based on those from the European Social Survey [40].

The participation criteria for LKI_2016 was being an immigrant (Immigrants were defined as individuals born abroad with two foreign-born parents and four foreign-born grandparents) aged 16 years and above, living in Norway with a minimum of 2 years’ residence by 1st October 2015, and originally from Poland, Bosnia and Herzegovina, Kosovo, Turkey, Iraq, Iran, Afghanistan, Pakistan, Sri Lanka, Vietnam, Eritrea and Somalia. The number of participants above retirement age (67 years) were very few, so we include only those up to age 66 years in our analyses. At the time of data collection, there were 214,000 immigrants from these countries living in Norway, making up almost one third of all immigrants [39].

From the randomly selected immigrants, groups of 500–900 individuals were sent an invitation letter with a brief description of the intent of the project, and address to respond (email or telephone) in case they were willing to participate (consent), and a brochure explaining the project every other week [41]. In recognition of their participation all participants were offered a gift card worth 300 kroners (30 Euros). If the participants were willing to participate in the survey, they responded either by email or phone and indicated the channel they would prefer for the interview.

The data in LKI_2016 was collected by computer-assisted telephone, or face-to-face interviews at a place of the participants’ choice. Data collection was conducted between November 2015 and July 2016. Eighteen percent of the interviews were conducted as personal interviews, and 82% were over the telephone. Interviews were available in each of the 12 countries main language(s) in addition to English. More detailed description of sampling and data collection is available in the report from Statistics Norway [39].

### Sample

A total of 8156 immigrants, aged 16–74 years were randomly selected from the population register on 1.10.2015. Of this, 4435 immigrants (1971 women and 2368 men) from aforementioned 12 countries participated in the study, a response rate of 54.4%. However, less than 2% (*N* = 85) of the participants were over 67 years, too few when broken down by gender and country background. They were therefore removed from the sample. In addition, 156 participants did not report either their height or weight and as a result their Body Mass Index (BMI) could not be calculated, and as BMI is our main response variable we removed these participants from the sample, leaving 4194 participants (men *N* = 2334 and women *N* = 1860) in the analyses.

### Ethics

This dataset was collected by Statistics Norway and issued by the Norwegian Centre for Research Data. Ethical approval or consent from participants was not required for this study because the dataset was anonymized. We conducted the analyses in accordance with the Norwegian Centre for Research Data’s data protection regulations.

### Variables

BMI: respondents were asked to report their weight (without clothes and shoes) in kilograms and height (without shoes) in metres. If the respondent was pregnant she was asked to report her weight before pregnancy. According to the WHO definition, BMI was divided into four categories underweight (< 18.5), normal (18.5–24.9), over-weight (25.0–29.9) and obese (≥30.0) [42]. For the analysis, we collapsed the categories underweight and normal weight into one category because of few numbers in the underweight category. We further modified the BMI variable into a dichotomous variable “not overweight/obese” (BMI < 25) and “overweight/obese” (merging over-weight and obese categories) BMI ≥ 25. The following variables were used in the analyses gender, age-groups, marital and employment status, time of residence, self-reported diabetes and physical activity. They were categorized as; gender: men (0), women (1). Age-groups; 16–24 years, 25–44 years, 45–66 years. Country of origin; Poland, Turkey, Bosnia-Herzegovina, Kosovo, Eritrea, Somalia, Afghanistan, Sri-Lanka, Iraq, Iran, Pakistan and Vietnam. We used Vietnam as the reference category. Employment Status; employed/ not employed. Marital status; Cohabitant/Married, Single, Widowed/Divorced/Separated. Education; < 10 years, Elementary school (10 years), High school (13 years), University level. Length of residence; ‘less than 15 years’ and ‘more than 15 years’. Diabetes; Yes/No. Physical Activity; was defined as any activity (walk briskly, sports, swimming) for 30 min or longer. It was categorized as ‘never’, ‘less than once/week’, ‘once/week’.

### Statistical analysis

We used a combination of chi-square and t-tests to see the differences between the two groups (Not overweight/obese, overweight/obese). We ran several logistic regression [43] analyses to assess the association between BMI and various socio-demographic indicators among immigrants while controlling for the covariates [43]. We present the crude analysis (model 1), and model 2 with adjustments for gender, age group, country of origin, marital status, education, physical activity, residence time, self-reported diabetes and employment. We also ran regression analyses separately for men and women. We report significance levels, *p < 0.05* significant, *p < 0.001* highly significant and 95% confidence intervals. We used statistical software STATA version 15 for all the analysis.

## Results

Our study sample included 4194 immigrants from 12 different countries. Approximately 53% of the sample (both men and women) was overweight/obese. Table 1 presents the distribution of participant characteristics by overweight/obesity and associations between participant characteristics and overweight/obesity. There was a significant difference in overweight/obesity by gender. More men (60%) were overweight/obese as compared to women (44%). Participants aged between 45 and 66 years comprised only (33%) of the sample but had the highest proportion (68%) of overweight/obese individuals. The highest proportion of overweight/obese individuals (67%) were from Turkey, followed by Poland (63%) and the lowest (27%) were from Vietnam.

**Table 1:** Distribution of participant characteristics by overweight/obesity and associations (odds ratio (OR) and 95% confidence intervals (95% CI) between participant characteristics and overweight/obesity, *n* = 4194 immigrants in the 2016 Living Conditions Survey in Norway

	***Total n (%)***	***Overweight/obese*** ***BMI ≥ 25.0 kg/m***^***2***^	***Model 1 (crude)*** ***OR (95% CI)***	***Model 2 (adjusted)*** ***OR (95% CI)***
	4194	2221 (53.0)		
Gender				
Men	2334 (55.7)	1394 (59.7)	1	1
Women	1860 (44, 3)	827 (44.5)	0.5 (0.5–0.6)	0.5 (0.4–0.6)
**Age-groups**				
16–24	563 (13.4)	153 (27.2)	1	1
25–44	2251 (53.7)	1128 (50.1)	2.7 (2.2–3.3)	2.4 (1.9–3.1)
45–66	1380 (32.9)	940 (68.1)	5.7 (4.6–7.1)	4.3 (3.2–5.8)
**Country of origin**				
Vietnam	329 (7.8)	90 (27.4)	1	1
Turkey	341 (8.1)	228 (66.7)	5.4 (3.8–7. 5)	5.9 (4.2–8.4)
Kosovo	362 (8.6)	227 (62.7)	4.5 (3.2–6.2)	5. 9 (4.2–8.3)
Poland	365 (8.7)	229 (62.7)	4.5 (3.2–6.2)	5.6 (4.0–8.1)
Iraq	340 (8.1)	201 (59.1)	3.8 (2. 8–5.3)	5.4 (3.8–7.7)
Pakistan	292 (6.9)	192 (65.8)	5.1 (3.6–7.2)	5.0 (3.5–7.2)
Bosnia	345 (8.2)	208 (60.3)	4.0 (2.9–5.6)	4.6 (3.2–6.4)
Somalia	344 (8.2)	168 (48.8)	2.5 (1.8–3.5)	4.1 (2.9–6.0)
Afghanistan	345 (8.2)	147 (42.6)	2.0 (1.4–2.7)	3.7 (2.6–5.4)
Iran	381 (9.1)	196 (51.4)	2.8 (2.0–3.8)	3.4 (2.4–4.8)
Sri Lanka	373 (8.9)	201 (53.9)	3.1 (2.3–4.3)	2.8 (2.0–3.9)
Eritrea	377 (9.0)	134 (35.5)	1.5 (1.1–2.0)	2.1 (1.5–3.0)
**Marital status**				
Single	1017 (24.2)	352 (16.0)	1	1
Cohabitant/Married	2792 (66.6)	1650 (74.3)	2.7 (2.3–3.2)	1.6 (1.3–1.9)
Widowed/Divorced/Separated	382 (9.1)	219 (10.0)	2.5 (2.0–3.2)	1.5 (1.1–2.0)
**Education**				
< 10 years	812 (19.4)	436 (53.6)	1	1
10 years	1443 (34.4)	724 (50.2)	0.9 (0.7–1.0)	1.0 (0.9–1.3)
13 years	974 (23.2)	557 (57.2)	1.1 (1.0–1.4)	1.0 (0.8–1.3)
University	975 (23.2)	504 (51.7)	0.9 (0.8–1.1)	0.8 (0.7–1.0)
**Length of residence**				
<= 15 Years	2569 (61.2)	1263 (49.1)	1	1
> 15 years	1625 (38.8)	958 (59.0)	1.5 (1.3–1.7)	1.1 (0. 9–1.4)
**Self-reported diabetes**				
No	3964 (94.5)	2042 (51.5)	1	1
Yes	222 (5.5)	179 (81.0)	3.9 (2.8–5.5)	2.4 (1.7–3.4)
**Physical Activity/week**				
Never	794 (19.0)	450 (57.0)	1	1
< Once	939 (22.4)	511 (54.4)	0.9 (0.7–1.1)	1.0 (0.8–1.2)
Once or more	2458 (58.6)	1258 (51.2)	0.8 (0.7–0.9)	0.9 (0.8–1.1)
**Employment Status**				
Employed	2717 (64.8)	1417 (52.1)	1	1
Not employed	1469 (35.2)	801 (54.2)	1.1 (1.0–1.2)	1.2 (1.1–1.4)

Almost three-quarters of married individuals were overweight/obese (74%) and 81% of self-reported diabetic participants were overweight/obese compared to 51% of non-diabetics. There was little variation in overweight/obesity by education and physical activity.

In both the crude and adjusted models (Table 1), men had twice the odds of being overweight/obese as compared to women. The odds of being overweight/obese increased with age. Immigrants from Turkey had 5.9 times the odds (CI; 4.2–8.4) of being overweight/obese as compared to those from Vietnam, followed by immigrants from Kosovo, Poland, Iraq and Pakistan. Those who were married/cohabiting or divorced/separated or widowed had higher odds of being overweight/obese as compared to never married participants. Similarly, those who were unemployed were 10% more likely to be overweight/obese as compared to employed immigrants. Participants with self-reported diabetes were 2.4 times more likely to be overweight/obese compared to those without diabetes. We did not find any significant relationship between overweight/obesity and education, physical activity or length of residence in Norway.

We also ran regression analyses separately for men and women, and present the results of only the adjusted analyses in Table 2. The odds for overweight/obesity were greatest in Somali, Iraqi and Pakistani women compared with Vietnamese women who were the least overweight/obese. Whereas odds for overweight/obesity were greatest in men from Turkey, Poland, Bosnia and Kosovo. Age was an important factor for both men and women with the oldest group (45–66 years) having the highest odds of overweight/obesity as compared to the youngest (16–24 years). While physical activity was not related to overweight/obesity for men, women exercising at least once a week had a 24% lower chance of being overweight/obese. The odds for being overweight/obese were 5.1 (CI; 2.6–10.0) in self-reported diabetic women as compared to non-diabetic women. Women who were not employed had a 31% higher chance of being overweight/obese as compared to women who were employed.

**Table 2:** Associations (odds ratio (OR) and 95% confidence intervals (95% CI) between participant characteristics and overweight/obesity stratified by gender

	Men		Women
	Odds Ratio (95% CI)		Odds Ratio (95% CI)
**Age-groups**		**Age-groups**	
16–24	1	16–24	1
25–44	2.9 (2.3–3.8)	25–44	2.9 (1.9–4.5)
45–66	5.2 (3.3–6.8)	45–66	6.4 (4.0–10.4)
**Country of origin**		**Country of origin**	
Vietnam	1	Vietnam	1
Turkey	5.2 (3.2–8.3)	Somalia	13.1 (7.4–23.1)
Poland	4.2 (2.7–6.1)	Iraq	8.6 (4.9–14.9)
Bosnia	4.1 (2.6–6.5)	Pakistan	7.5 (4.2–13.4)
Kosovo	3.9 (2.5–6.1)	Kosovo	7.0 (4.1–12.1)
Pakistan	3.5 (2.2–5.5)	Turkey	6.8 (4.0–11.6)
Iraq	2.7 (1.7–4.2)	Poland	5.7 (3.2–10.2)
Iran	2.4 (1.5–3.6)	Afghanistan	5.5 (3.0–10.1)
Sri Lanka	2.1 (1.3–3.2)	Bosnia	4.8 (2.8–8.2)
Afghanistan	1.2 (0.8–1.8)	Sri Lanka	4.5 (2.7–7.6)
Somalia	1.0 (0.6–1.6)	Iran	4.3 (2.5–7.3)
Eritrea	0.9 (0.6–1.4)	Eritrea	3.8 (2.2–6.5)
**Marital status**		**Marital status**	
Single	1	Single	1
Cohabitant/Married	3.0 (2.5–3.7)	Cohabitant/Married	1.9 (1.4–2.6)
Widowed/Divorced/Separated	2.4 (1.7–3.5)	Widowed/Divorced/Separated	1.9 (1.3–2.9)
**Education**		**Education**	
< 10 years	1	< 10 years	1
10 years	0.9 (0.7–1.7)	10 years	1.1 (0.8–1.5)
13 years	1.4 (1.1–1.8)	13 years	1.1 (0.76–1.5)
University	1.3 (1.0–1.7)	University	0.7 (0.5–1.0)
**Length of residence**		**Length of residence**	
<= 15 Years	1	<= 15 Years	1
> 15 years	1.2 (0.9–1.5)	> 15 years	1.1 (0.9–1.5)
**Self-reported diabetes**		**Self-reported diabetes**	
No	1	No	1
yes	2.4 (1.6–3.6)	yes	5.1 (2.6–10.0)
**Physical Activity/week**		**Physical Activity/week**	
Never	1	Never	1
< Once	1.1 (0.9–1.5)	< Once	1.0 (0.7–1.3)
Once or more	1.2 (0.9–1.5)	Once or more	0.8 (0.6–0.9)
**Employment Status**		**Employment Status**	
Employed	1	Employed	1
Not employed	1.1 (0.9–1.4)	Not employed	1.3 (1.0–1.6)

## Discussion

This study assessed the relationship between overweight/obesity and socio-demographic factors among immigrants in Norway. The main socio-demographic factors identified were age, gender, country of origin and marital status. Having self-reported diabetes increased the odds of being overweight/obese. However, since the dataset is cross-sectional, we cannot establish whether overweight/obesity was due to self-reported diabetes or vice versa. We did not find any association between education, employment status, being physically active and overweight/obesity.

Age is a strong determinant of overweight/obesity in our study, which is in line with previous research [21, 44, 45]. The results also show higher overweight/obesity among immigrant men as compared to women. This higher obesity among men, is in line with the obesity trend in the general population in Norway [46, 47]. The study shows that Turkish, Polish, Bosnian, and Kosovan men have the highest odds of being overweight/obese even after adjustment for age, marital status, physical activity and employment status. This finding is similar to that of another study done in Australia, where they reported evidences of ethnic difference in overweight/obesity among males from North Africa/ Middle East [48]. In addition, data from all these countries indicate a rise in overweight/obesity among men [49, 50] . Furthermore, all these countries are located in Europe and the percentage of overweight/obese individuals among men has increased four times in Europe over the last few years [50, 51]. A literature review published in 2012, stated that in developed countries, more men are overweight than women. This may be the result of socio-cultural dynamics, gender differences in terms of consumption of food, and acculturation through complex socio-cultural pathways [52].

Our study also showed that the country of origin had a significant association with the odds of being overweight/obese. In Norway, immigrants come from over 200 cultural settings and prevention of overweight/obesity among the highly diverse immigrant communities is a challenge. This challenge partially stems from the fact that many immigrant communities have different concepts of health and health care than mainstream societies in the receiving country [53]. For example, a slim body is associated with poverty and ill health in many African cultures, while physical activity is perceived as something that is integrated into everyday lives; thus the promotion of leisure time physical activity such as joining a fitness class or going for a long walk or jogging is not considered as useful or necessary [54]. In some communities, activities involving sweating and deep breathing are associated with negativity, whereas sitting and resting are associated with affluence. This cultural practice might have affected the results of our study and thus we did not find an overall association between physical activity and overweight/obesity in our sample.

However, when we stratified our analyses by gender we observed an association (OR: 0.7: CI; 0.6–0.9) of physical activity with the odds of being overweight/obese, among women who engaged in physical activity once or more per week. This may be a chance (random error) finding. A fundamental problem in making a causal interpretation of associations from observational data is the possibility that such associations are due to confounding. Although we did adjust our estimates for confounders, we cannot exclude the possibility of residual confounding, as we had to rely on the information provided by the participants. Thus we cannot rule out the possibility of over reporting and possible misclassification of the physical activity variable. Furthermore, lack of data on dietary intake in our sample precludes us from drawing any conclusion based on the association between diet and physical activity. However, it is well-known that immigrants may have an increasingly unhealthy diet after migration to HIC, but their diet is generally healthier than native populations, so this is unlikely to be a major contributor to increased obesity risk [13]. Our results demonstrate an association between physical activity and overweight/obesity among women but not for men, however we are unable to support this finding from published studies due to paucity of relevant literature.

We did not find an association between the length of residence and being overweight/obese. Although, published studies show that with the passage of time, immigrants gain more weight [15, 55–57]. Higher rates of obesity among some groups of immigrants in Norway may be explained by immigrants’ adaptation to an unhealthy lifestyle. Evidence presented across multiple studies indicate an association between acculturation, measured by duration of stay in the receiving country and obesity [20, 57, 58]. Gele and Mbalilaki, who estimated the prevalence of obesity among African immigrants in Norway, found that the obesity pattern increased with the duration of stay in Norway, as evidenced by a higher BMI rate beginning after 5 years [21]. As stated before, immigrant health deteriorates and BMI increases as time since migration increases, and this does not become evident before 10–15 years [20, 59, 60], so the cut-off 15 years residence time in our study seems reasonable. However, it is difficult to determine if this increase in BMI is due to unhealthy acculturation or it would have simply increased even if immigrants remained in their country of origin [60].

Our results showed that Somali women had the highest likelihood of being overweight/obese. According to the WHO statistics (2008), obesity is rare in Somalia; approximately 3.1% of women and 6.4% of men were overweight/obese in 2008. Similarly, Somalia has the lowest prevalence of self-reported diabetes in Arab League countries [21]. Nevertheless, prior studies on obesity among Somali women have warned on increasing obesity among them with increased years of living in Norway [21, 61]. Research shows that the largest proportions of modifiable risk for self-reported diabetes among Somali women were contributed by high BMI and high waist circumference, which coincides with high prevalence of sedentary lifestyle [61]. Increased obesity among Somali women may be due to the limited access to a tailored physical exercise structure and, most importantly, a lack of proper health information to help them acquire adequate physical exercise [62].

A prior study highlighted the challenges that Somali women face in becoming physically active, which include time pressure, a lack of financial affordability for training facilities, and an absence of a tailored physical activity environment. Next to Somali women, women from Pakistan, Iraq, Turkey and Kosovo also have high odds of being overweight/obese. The overweight/obesity among women from Somalia, Pakistan and Iraq concords with global inactivity prevalence. In Eastern Mediterranean region, Somalia, Pakistan and Iraq, had the highest rates of inactivity among WHO regions, approaching 50% of women and 40% of men [37].

However, it would not be justifiable just to label physical inactivity as the lone cause of overweight/obesity. It is known that numerous factors influence body weight. Some of these factors such as dietary intake, physical activity, social factors can be individually controlled, whereas factors like developmental determinants, genetic makeup, gender, and age are out of individual control [63]. In case of overweight/obesity among Somali women, a probable explanation could be that in addition to their traumatic experiences in decades long civil war, they face a number of challenges, such as language barriers, discrimination, poor understanding of country’s health system, and religious differences when adapting to their new country. This in turn leads to, an increase in sedentary behavior, coupled with a drastic change in diet and ultimately leading to cardiovascular risk, overweight, and diabetes [64].

Another important factor worth mentioning with regards to increased levels of overweight/obesity is low level of health literacy among many groups of immigrants [65]. A previous study reported low levels of health literacy among Somali women and 71% of them lacked the ability to obtain, understand and act upon health information and services, and to make appropriate health decisions [66]. Similarly, another study from Sweden reported, health literacy to be just 60%, and factors such as being born in Somalia were associated with an increased risk of having an inadequate health literacy [67]. Therefore, having inadequate health literacy may also be responsible for the unhealthy behavior among immigrants.

Our study has some major limitations. An important limitation is its cross-sectional design, hence making it difficult to establish the causes. Moreover, most of the variables including weight and height were self-reported, with a distinct possibility of both under- or over-reporting. Research suggests that individuals have tendency to under-report their weight, and the size of the discrepancy between self-reported and actual weight increases with increasing body weight [68]. This may result in an underestimation of the number of individuals who are overweight/obese. However, the same is true for over-reporting as well. This points to the fact that we need more research to confirm the presence and direction of biases in weight-reporting among adults in different immigrant groups [69, 70]. Self-reported diabetes is another limitation of the study as up to 50% of individuals with diabetes in Norway are not aware of their diabetes status [71]. Therefore, self-reported diabetes is simply not a representation of adults with diabetes in Norway. Another limitation is that the low number of participants from the different immigrant communities might not be completely representative of immigrants as a whole in Norway, thus the findings may not be generalizable. The study also lacks detailed lifestyle questions. For instance, although dietary intake is a strong determinant of the body weight, data on neither caloric intake nor fruit and vegetable intake was available. Finally, collapsing the categories of overweight and obesity into one might have mitigated the estimates. However as previously mentioned, the dichotomous variable for overweight/obesity was per WHO cut offs (BMI > 25 kg/m^2^) [72]. In addition, overweight/obesity as a combined category is clinically relevant due to the established health consequences of exceeding a body mass index of 25 kg/m^2^ [73]. Furthermore, it is also a policy-relevant categorization reflecting international obesity reduction targets and indicators [72].

Despite these limitations, the study provides important information on overweight and obesity of different under-researched immigrant groups, in addition to highlighting specific groups that are at high risk of overweight/obesity and subsequent CVD. The high proportion of certain immigrant groups with obesity will generate considerable obesity-related costs owing to increased health care costs as well as productivity loss. This probable epidemic will also reduce the quality and duration of life in this population.

## Conclusions

Our findings show that there is heterogeneity among different immigrant groups. Therefore to meet immigrant’s preventive health needs, particularly obesity prevention, the host countries need a greater understanding of the health status, health behaviors, and risk factors prevalent in different ethnic groups.

In addition, our study suggests the need for culturally adapted prevention strategies targeting immigrant groups with high rates of obesity. Moreover, the findings of this study call attention to the importance of a greater understanding of the processes leading to obesity among different immigrant groups in Norway. Groups such as Somali, Pakistani, Kosovo and Iraqi women, and men from Turkey, Poland, Bosnia and Kosovo should be targeted.

## Data Availability

The data is available from NSD (Norwegian Centre for Research Data) upon request.

## Abbreviations

CVD: Cardiovascular disease
HICs: High-income countries
LMIC: Low- and middle-income countries
NIPH: Norwegian Institute of Public Health
BMI: Body Mass Index
WHO: World Health Organization
